# A new technique for surgical haemorrhoidectomy without post-operative complication: A case series

**DOI:** 10.1016/j.amsu.2022.103467

**Published:** 2022-03-14

**Authors:** Imam Sofii, Handy Darmawan, Fauzan Kurniawan, Ahmad Shafa Hanif, Reagan Resadita, Amelia Sophia Ramadhini

**Affiliations:** Digestive Surgery Division, Department of Surgery, Faculty of Medicine, Universitas Gadjah Mada/Dr. Sardjito Hospital, Yogyakarta, 55281, Indonesia

**Keywords:** Anal dilatation, Hemorrhoid, Hemorrhoidectomy, Radial sutures, Triangle incision

## Abstract

**Introduction:**

Hemorrhoids are a common coloproctology problem and among 10% of cases need surgical intervention. However, the established surgical interventions still have many complications.

**Case presentation:**

We reported three female patients, who presented with circular 3rd degree internal hemorrhoids. The surgical treatment was performed with pre-operative anal dilatation using a 33 mm dilator for 20 minutes, followed by triangle incision above the dentate line. The hemorrhoid excision was performed, and the wound was sutured with simple interrupted radial sutures using a multifilament absorbable 3-0 thread. There were neither complaints of pain, bleeding, anal incontinence, anal stenosis, wound dehiscence, nor recurrence at the first, second, and fourth weeks of follow-ups in all patients.

**Discussion:**

Post-operative bleeding, pain, and anal incontinence are common after an open hemorrhoidectomy, while suture breakage and anal stenosis were reported after the old technique of closed hemorrhoidectomy. Stapled hemorrhoidectomy had less complications but requires a relatively more expensive cost for the device itself. We performed a combination of preoperative anal dilatation, above dentate line triangle incision, and simple interrupted radial sutures to treat the patients with 3rd degree internal hemorrhoids, which resulted in no post-operative complications within the first month of follow-up.

**Conclusion:**

A combination of preoperative anal dilatation, above dentate line triangle incision, and radial suture technique is a simple and effective surgical option for treating a 3rd degree hemorrhoid.

## Highlights

• Surgical hemorrhoidectomy indicated for third- and fourth-degree hemorrhoid.
• Conventional surgical hemorrhoidectomy had many post-operative complications.
• A combination of preoperative anal dilatation, above dentate line triangle incision, and radial suture has favorable outcome.

## 1 Introduction

Hemorrhoid is a common disease, for which 10% of cases require surgical intervention. In cases with 3rd and 4th degree hemorrhoids, surgical intervention with various modalities is the treatment of choice [1,2]. There are several post-operative complications related to surgical hemorrhoidectomy, which are anal stenosis, wet anus, anal incontinence, bleeding, and severe pain [[3], [4], [5]]. Here, we report three cases of 3rd degree internal hemorrhoids in Dr. Sardjito Academic Hospital, which were successfully treated with a combination of preoperative anal dilatation, above dentate line triangle incision, and radial suture technique. In addition, there were no pain, bleeding, nor other post-operative complications found after 1 month of follow-up. This report is presented in line with the PROCESS 2020 guideline [6].

## 2 Presentation of cases

We report 3 cases of 3rd degree internal hemorrhoid. All the patients had normal preoperative laboratory examination result and had undergone preoperative colon preparation using laxative suppository. The procedures were performed by a board-certified senior digestive surgeon with 12 years of experience in coloproctology field.

## 3 Case 1

**Fig. 1:**
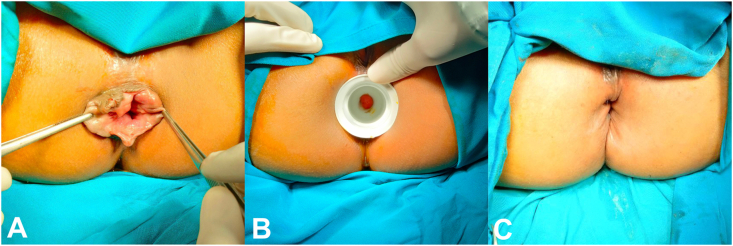
Case 1 (A) before haemorrhoidectomy, (B) during dilatation, (C) after haemorrhoidectomy.

A 43-year-old female presented with rectal bleeding and painless anal mass, which could not be reduced spontaneously (Fig. 1). She reported an intermittent hematochezia without changes in frequency, consistency and caliber of bowel movements. In the physical examination, there were circular hemorrhoid piles found protruding from the anal canal, which still could be reduced manually. Therefore, surgical hemorrhoidectomy was chosen for the treatment of the patient.

**Fig. 2:**
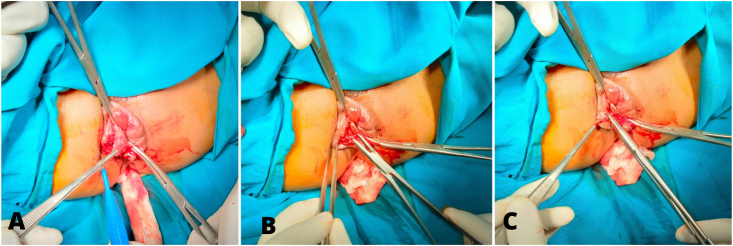
(A) Triangle incision, (B) haemorrhoidal excision, (C) radial suture.

Anal dilation was performed initially using a 33 mm dilator for 20 minutes under spinal anesthesia. The circular hemorrhoidal piles were identified, and the triangle incision was made above the dentate line. The size of the triangle was determined according to the size of the protruding piles. Furthermore, the hemorrhoidal piles were excised, and the wounds were sutured radially by a simple interrupted suture. These procedures were performed for the excision of piles at 3, 7, and 11 o'clock. The closure of the wounds was performed using a multifilament absorbable suture 3-0 (Fig. 2). There was no residue of hemorrhoidal tissue nor bleeding found in the evaluation after wound suturing. The surgical wound was covered by an anal tampon for the first 24 hours. After surgery, the patient was treated with intravenous analgesia and antibiotics for the first 24 hours, which were switched to the oral regimen the day after surgery. At the postoperative outpatient follow-up in the first, second and the fourth weeks, the patient reported no pain, no bleeding, no anal incontinence, nor recurrence of her previous complaints. In addition, anal stenosis, wound dehiscence, and hemorrhoidal residue was not found at the physical examination.

## 4 Case 2

**Fig. 3:**
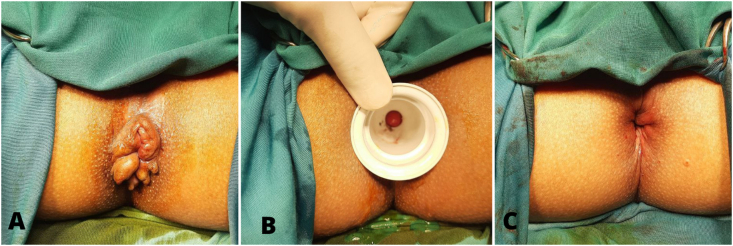
Case 2 (A) before haemorrhoidectomy, (B) during dilatation, (C) after haemorrhoidectomy.

A 24-year-old female presented with postpartum hemorrhoids, which could not be reduced spontaneously (Fig. 3). She reported no bleeding and no pain from the hemorrhoid piles. On the physical examination, there were circular hemorrhoidal piles found protruding from the anal canal. Therefore, surgical hemorrhoidectomy was chosen for the treatment of the patient. The patient underwent the same surgical procedure and post-operative treatment as the first patient. At the postoperative outpatient follow-up in the first, second and the fourth weeks, the patient reported no pain, no bleeding, no anal incontinence, nor recurrence of her previous complaints. Furthermore, anal stenosis, wound dehiscence, and hemorrhoidal residue were not found at the physical examination.

## 5 Case 3

**Fig. 4:**
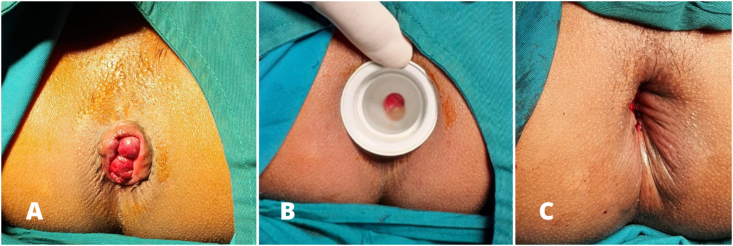
Case 3 (A) before haemorrhoidectomy, (B) during dilatation, (C) after haemorrhoidectomy.

A 30-year-old female presented with anal mass, which must be reduced manually (Fig. 4). She reported no pain from the mass with intermittent bleeding upon defecation. On the physical examination, there were circular hemorrhoidal piles found protruding from the anal canal. Surgical hemorrhoidectomy with anal dilatation was performed. The patient underwent the same surgical procedure and post-operative treatment as the previous reported patients. At the postoperative outpatient follow-up in the first, second and the fourth weeks, the patient reported no pain, no bleeding, no anal incontinence, nor recurrence of her previous complaints.

## 6 Discussion

The open hemorrhoidectomy (Milligan-Morgan) technique is the most common and effective surgical technique for treating hemorrhoids [3]. However, it has several reported complications due to its open wound, which are pain, wet anus, anal incontinence, fistula, abscess, bleeding, itching, urinary retention, incontinence symptoms, and prolonged wound healing [3,4,7]. In contrast, the closed hemorrhoidectomy technique (Ferguson) has better outcomes in reducing postoperative pain, bleeding, and time of recovery [8]. It was reported due to the closure of the wound using an absorbable running suture, which stops the bleeding and promotes faster primary wound healing. Unfortunately, this older closed hemorrhoidectomy technique has a high rate of suture breakage during the bowel movement [3].

Stapled hemorrhoidectomy is the most recent option for surgical hemorrhoidectomy, which has less post-operative pain, itching, and burning sensations. It is because this technique does not involve dissection and excision of the perianal skin [3,9]. The most common complication of this technique was early bleeding secondary from the arteriolar along the staple line. In addition, anastomotic dehiscence cases were reported due to malfunctioning staplers, surgical inexperience, and double ﬁring of the stapler. Stapled hemorrhoidectomy and Milligan-Morgan procedure have a similar rate of recurrence [9]. The recurrence after stapled hemorrhoidectomy was reported due to incomplete piles excision. The difficulty in identification of the amount of mucosa to be removed was the reason behind incomplete pile excision. This technique also needs extra funding for its stapler device, which is expensive [3].

**Fig. 5:**
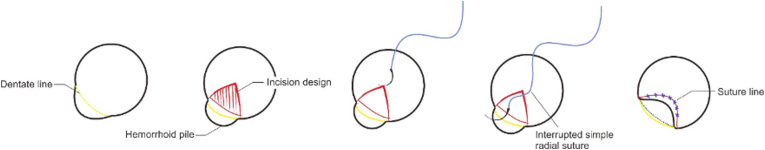
Schematic design of the entire procedures.

In this report, the wound closure was performed using a simple interrupted suture, which can prevent suture breakage, stop the bleeding effectively, and secure the wound healing process (Fig. 5). The suture was performed radially to prevent mucosal prolapse and recurrence of the complaint. Preoperative anal dilatation was performed to reduce the hypertonicity of the internal anal sphincter to prevent stenosis and reduce post-operative pain [10,11]. The incision superior to dentate line also ensures less post-operative pain due to the lack of afferent nerve fiber in it [12]. In the best of our knowledge, this procedure has never been performed before. In addition, it can also be performed by general surgeon in rural area due to its simplicity in both surgical material and technique. However, it still needs further study using a randomized control trial design with longer follow-up, more subjects, and more assessments for the different post-operative outcomes. This report had been registered to research registry, with UIN: 7686, can be accessed with this link: https://www.researchregistry.com/browse-the-registry#home/registrationdetails/6219c51b68d5d2001e192d26/

## 7 Conclusions

A combination of preoperative anal dilatation, above dentate line triangle incision, and radial suture technique is a simple and effective surgical option for treating a 3rd degree hemorrhoid. However, further trial study with more subjects is needed to establish this new technique.

## Ethical approval

The informed consent form was declared that patient data or samples will be used for educational or research purposes. Our institutional review board also do not provide an ethical approval in the form of case report. This trial already accepted by ethical committee of faculty of medicine, public health and nursing Universitas Gadjah Mada, with reference number KE/FK/0083/EC/2022.

## Sources of funding

The authors declare that this study had no funding source.

## Author contribution

Imam Sofii conceived the study and approved the final draft. Handy Darmawan and Amelia Sophia Ramadhini, drafted the manuscript. Ahmad Shafa Hanif, Fauzan Kurniawan dan Reagan Resadita critically revised the manuscript for important intellectual content. Imam Sofii, Handy Darmawan, Fauzan Kurniawan, Reagan Resadita, Amelia Sophia Ramadhini, and Ahmad Shafa Hanif facilitated all project-related tasks.

## Consent

Written informed consent was obtained from the patient for publication of this case report and accompanying images. A copy of the written consent is available for review by the Editor-in-Chief of this journal on request.

## Registration of research studies

Research Registry UIN: 7686 https://www.researchregistry.com/browse-the-registry#home/registrationdetails/6219c51b68d5d2001e192d26/

## Guarantor

Imam Sofii.

## Provenance and peer review

Not commissioned, externally peer reviewed.

## Declaration of competing interest

No potential conflict of interest relevant to this article was reported.
